# Adventures and Advances in Time Travel With Induced Pluripotent Stem Cells and Automated Patch Clamp

**DOI:** 10.3389/fnmol.2022.898717

**Published:** 2022-06-22

**Authors:** Kadla R. Rosholm, Beatrice Badone, Stefania Karatsiompani, David Nagy, Fitzwilliam Seibertz, Niels Voigt, Damian C. Bell

**Affiliations:** ^1^Sophion Bioscience A/S, Ballerup, Denmark; ^2^Sophion Bioscience Inc., Woburn, MA, United States; ^3^Institute of Pharmacology and Toxicology, University Medical Center Göttingen, Göttingen, Germany; ^4^German Center for Cardiovascular Research (DZHK), Göttingen, Germany; ^5^Cluster of Excellence “Multiscale Bioimaging: from Molecular Machines to Networks of Excitable Cells” (MBExC), University of Göttingen, Göttingen, Germany

**Keywords:** hiPSC, stem cells, automated patch clamp, cardiomyocytes, neurons, ion channels

## Abstract

In the Hollywood blockbuster “The Curious Case of Benjamin Button” a fantastical fable unfolds of a man’s life that travels through time reversing the aging process; as the tale progresses, the frail old man becomes a vigorous, vivacious young man, then man becomes boy and boy becomes baby. The reality of cellular time travel, however, is far more wondrous: we now have the ability to both *reverse and then forward time* on mature cells. Four proteins were found to rewind the molecular clock of adult cells back to their embryonic, “blank canvas” pluripotent stem cell state, allowing these pluripotent stem cells to then be differentiated to fast forward their molecular clocks to the desired adult specialist cell types. These four proteins – the “Yamanaka factors” – form critical elements of this cellular time travel, which deservedly won Shinya Yamanaka the Nobel Prize for his lab’s work discovering them. Human induced pluripotent stem cells (hiPSCs) hold much promise in our understanding of physiology and medicine. They encapsulate the signaling pathways of the desired cell types, such as cardiomyocytes or neurons, and thus act as model cells for defining the critical ion channel activity in healthy and disease states. Since hiPSCs can be derived from any patient, highly specific, personalized (or stratified) physiology, and/or pathophysiology can be defined, leading to exciting developments in personalized medicines and interventions. As such, hiPSC married with high throughput automated patch clamp (APC) ion channel recording platforms provide a foundation for significant physiological, medical and drug discovery advances. This review aims to summarize the current state of affairs of hiPSC and APC: the background and recent advances made; and the pros, cons and challenges of these technologies. Whilst the authors have yet to finalize a fully functional time traveling machine, they will endeavor to provide plausible future projections on where hiPSC and APC are likely to carry us. One future projection the authors are confident in making is the increasing necessity and adoption of these technologies in the discovery of the next blockbuster, this time a life-enhancing ion channel drug, not a fantastical movie.

## Introduction

When Takahashi and Yamanaka discovered four genes (*c-Myc, Oct3/4, Sox2*, and *Klf4*), whose protein products were shown to reprogram (induce) somatic cells into pluripotent stem cells (Takahashi and Yamanaka, 2006), these Yamanaka factors were heralded as a breakthrough in biomedical research (Press release, 2012). For the first time, pluripotency, the ability to form a range of specialized cell types from a pluripotent cell, was possible from mature, adult somatic cells, no longer requiring embryonic stem cells (Shi et al., 2017; Yoshida and Yamanaka, 2017).

The limited availability of embryonic tissue to derive embryonic stem cells coupled with legal and ethical regulations, always stymied stem cell research development and progress (Lo and Parham, 2009; Jha, 2011; Moradi et al., 2019). With the discovery of the Yamanaka factors, somatic cells could be taken from adult tissue and reprogrammed, turning back the clock on mature cells to an embryonic-like, pluripotent state. The processes where pluripotent stem cells (PSCs) are differentiated and matured, allows fast-forwarding of cells into specialized cell lines providing model cells for *in vitro* research (Rowe and Daley, 2019).

The use of human induced pluripotent stem cells (hiPSCs) has a number of advantages: it reduces the use of animal tissue; compared to cells overexpressing recombinant protein [e.g., Chinese hamster ovary (CHO)] human cells are more physiologically relevant and translatable models; in comparison with primary cells, such as tissue dissociated cardiomyocytes and neurons, they are easier to access (typically from a blood or skin sample) and to maintain in culture; hiPSCs can be derived from both healthy and patient populations, providing both human “wild type” control and diseased models; and, since hiPSCs can be derived from any of us, the options to study a personal genotype and phenotype are broad, allowing for truly personalized (also termed precision or stratified) medicines and interventions (Matsa et al., 2016; Hamazaki et al., 2017; Doss and Sachinidis, 2019). This technology, however, has limitations worth considering, such as: the fetal nature of hiPSCs; the lifespan in culture; the long-term maturation process (for details on the challenges and limitations see the following section “Maturity and Subtype Specificity of hiPSC-Derived Cell Lines”).

Whilst these seminal developments in generating and differentiating hiPSCs were taking place, similar strides were being made in ion channel recording techniques. The dazzlingly brilliant but highly time consuming and technically challenging manual patch clamp (MPC) technique (Hamill et al., 1981) was simplified, made faster and more efficient, through a combination of planar patch clamp and automation (Bell and Dallas, 2018). By switching from manual micro-manipulation of a single recording electrode down onto an adherent cell in MPC, to the application of cell suspensions by robot pipettors to arrays of electrodes in automated patch clamp (APC), the technical hurdles were lowered whilst vastly increasing throughput of ion channel recordings.

Thus, with hiPSCs and APC, researchers now have the capabilities to characterize ion channel populations in hiPSCs and their differentiated cell lines, for instance exploring the effect of different reprogramming, differentiation and maturation conditions and introducing quantifiable metrics for quality assessment. Furthermore, as the hiPSC technology develops, APC will be applicable for safety pharmacology and drug screens in highly translatable hiPSC model cell lines from healthy and disease patient populations.

In this review, the authors aim to describe advances in the generation of mature hiPSC-derived cell lines; developments in cell suspension preparation for APC measurements; APC techniques and technologies that have made these once challenging ion channel recordings more successful, physiologically relevant, and amenable to high throughput screening; and the key challenges solved thus far and those to be faced. Finally, we provide examples of novel applications of hiPSCs and APC. These techniques offer huge potential to advance biomedical research and drug discovery, and the future they project looks to be bright and filled with many novel and exciting findings.

## Reprogramming, Differentiation, and Maturation of Human Induced Pluripotent Stem Cell Derived Cell Lines

During the last decade, substantial effort has gone into developing protocols for generating specialized hiPSC-derived cells and tissues for applications within a range of areas, such as blood, smooth muscle, liver, heart, and brain (Bellin et al., 2012; Shi et al., 2017; Rowe and Daley, 2019).

**FIGURE 1:**
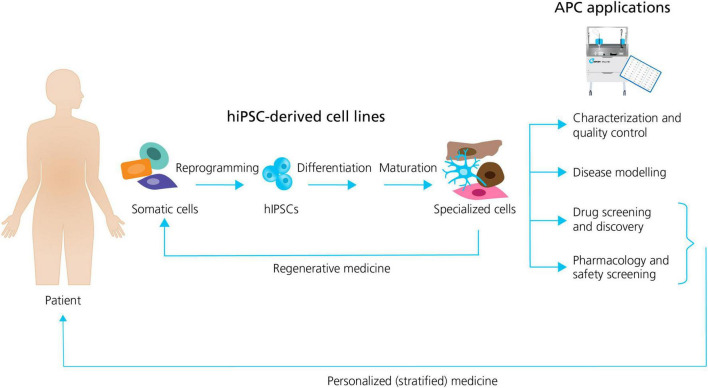
Generation of specialized hiPSC-derived cells and APC applications. Somatic patient cells can be reprogrammed into hiPSCs, followed by the differentiation and maturation into specialized cells. The specialized cells can be used directly in regenerative medicine, or they can be employed together with APC for a range of applications including: characterization and quality control; disease modeling; drug screening and discovery; and pharmacology and safety screening. Patient hiPSCs can be used as personalized drug screening and pharmacology models in trials. Adapted from Bellin et al. (2012), with permission from publisher Springer Nature.

In general, the formation of different specialized hiPSC derived cells follow the same sequence of events: (1) the reprogramming (induction) of human unipotent somatic cells into hiPSCs by directed expression of certain transcriptional factors; and, (2) the differentiation and maturation of hiPSCs into specialized cells, typically by using signaling pathway agonists and antagonists in specific concentrations to induce stepwise progression through the embryonic development stages (Bellin et al., 2012; Figure 1).

In this section we aim to summarize the latest methodological advances of hiPSC generation and differentiation and discuss the key challenges in the field.

### Genetic Variability of Human Induced Pluripotent Stem Cell Derived Cell Lines

A challenge with hiPSC-derived cell lines is the genetic variability existing within and between hiPSC populations, and an ongoing task is to control and maintain genome integrity during reprogramming and differentiation (Assou et al., 2018; Popp et al., 2018). An important development in this regard was the introduction of non-integrating methods, such as *Sendai* virus (Fusaki et al., 2009), for introducing reprogramming factors, which aim to reduce the risk of variability or spurious gene activation/inactivation in hiPSC-derived cell lines (Shi et al., 2017; Berry et al., 2019).

Another focus is the inherent biological variability between donors stemming from their individual genetic and epigenetic background (Berry et al., 2019; Rowe and Daley, 2019), which has been estimated to be responsible for up to 46% of phenotype variability and also influences differentiation efficiency (Kilpinen et al., 2017). A strategy for circumventing inter-donor variability is to apply gene-editing technologies, such as CRISPR/Cas9 (Jehuda et al., 2018) to generate isogenic controls, which allow the investigation of disease-causing mutations with control cells having the same genetic background (Yoshida and Yamanaka, 2017).

### Maturity and Subtype Specificity of Human Induced Pluripotent Stem Cell-Derived Cell Lines

A further key challenge is to improve the maturity and subtype specificity of hiPSC-derived cell lines. Despite improved protocols for producing specific somatic cell types and subtypes, the vast majority of hiPSC-directed differentiations produce populations that most closely resemble fetal or neonatal cells (Berry et al., 2019), including the extensively studied hiPSC-derived cardiomyocytes (hiPSC-CMs; Kolanowski et al., 2017; Di Baldassarre et al., 2018; Ahmed et al., 2020), and hiPSC-derived neurons (hiPSC-neurons; Casalia et al., 2021; de Leeuw et al., 2021; Giacomelli et al., 2022). In both cases there is a further complexity in creating pure subtype populations, for example atrial or ventricular hiPSC-CMs (Cyganek et al., 2018; Feyen et al., 2020) or defining the desired hiPSC-neuron subtype (e.g., sensory, motor, or dopaminergic neurons; Davis-Dusenbery et al., 2014; Hartfield et al., 2014; Young et al., 2014).

**FIGURE 2:**
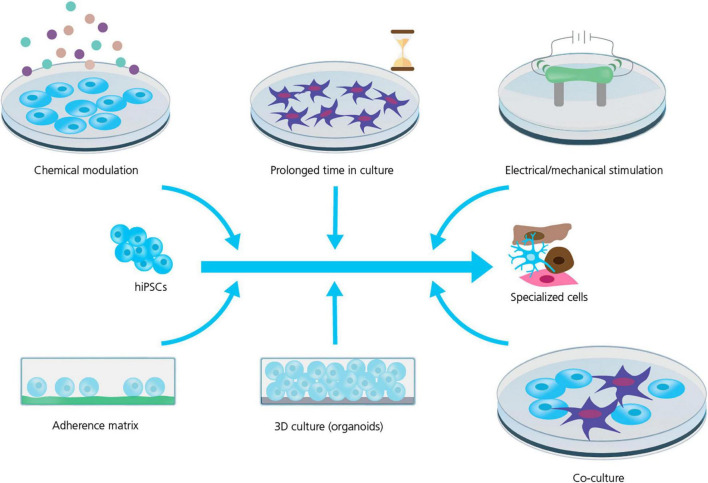
Strategies for improving hiPSC maturity. Several strategies are used to improve the maturity and phenotype specificity of hiPSC-derived cell lines. However, the mechanism and efficiency of these strategies are still being explored and requires rigorous characterization and quality control. Adapted from Ahmed et al. (2020), with permission under Creative Commons Attribution License (CC BY).

Strategies to improve cell maturation during differentiation are many (see Figure 2) and include: the addition of specific chemical cues during differentiation (Burridge et al., 2012; Yoshida and Yamanaka, 2017; Ahmed et al., 2020); replacing the energy source, for instance the addition of fatty acids as a substitute for glucose in the culturing media, has been shown to improve hiPSC-CM maturation by activating the required metabolic pathways (Sharma et al., 2015; Yoshida and Yamanaka, 2017; Yang et al., 2019); prolonged time in culture which does, however, also increase the risk of contamination and mutation accumulation (Blokzijl et al., 2016; Odawara et al., 2016; Breckwoldt et al., 2017; Yoshida and Yamanaka, 2017; Biendarra-Tiegs et al., 2019; Ahmed et al., 2020); electrical and mechanical stimulation (pacing) have been reported as valid methods to improve the maturity and function of hiPSC-CMs and -neurons (Ahmed et al., 2020; Giacomelli et al., 2022); and finally modulating the chemical and spatial microenvironment of the differentiating cell culture, for example changing the adherence matrix (Feaster et al., 2015; Depalma et al., 2021), cell culture geometry, e.g., using 3D culture (Lemoine et al., 2017; Tiburcy et al., 2017; Di Baldassarre et al., 2018; de Leeuw et al., 2021) or preparing co-cultures with supportive cells (Odawara et al., 2016; Andersen et al., 2020; Zafeiriou et al., 2020).

Cell reprogramming, differentiation and maturation are manipulations that lead to many intracellular changes that can affect the lifespan of cells. Indeed, a somatic cell obtained from a patient which is “rejuvenated” into a stem-cell like phenotype, shows intracellular signatures such as an active telomerase activity and immature mitochondria, as well as a decrease in senescence markers [e.g., senescence marker p21, (Strässler et al., 2018)]. After differentiation, although the cellular aging process may restart, certain improved characteristics remain compared to the parent cell (e.g., longer telomeres).

Due to these intracellular changes, additional challenges arise when referring to how the age of patients could influence the functionality and maturity of hiPSC-derived models and whether it is feasible to apply this technology in age-related diseases. There is also an increasing need to perform safety pharmacology in “aged” senescent cells to functionally model and translate the complex physiology, pathophysiology and pharmacology of the aging, senior population, particularly in light of the growth of this demographic (Fermini and Bell, 2022).

In addressing these challenges, there is a need to define quality standards during hiPSC reprogramming and differentiation, as well as rigorous and statistically sound characterization of the final product (Engle et al., 2018). The specific composition of ion channels is an important marker of cell maturity and subtype specificity, for example in hiPSC-and -neurons (Jonsson et al., 2012; Bardy et al., 2016; Goversen et al., 2018b), rendering APC important as a high-throughput (HT) characterization method providing ion channel maturation metrics. Thus, we envision molecular and genetic approaches in combination with APC electrophysiological characterization will generate authentic, translatable and quantifiable hiPSC models for disease investigation and drug-screening.

## Electrophysiological Measurements of Human Induced Pluripotent Stem Cell Derived Cell Lines Using Automated Patch Clamp

Ion channels represent attractive targets in hiPSC-derived cell lines, making electrophysiological studies of hiPSC-derived cell lines important not only for characterization (as mentioned above), but also for advances in biomedical research including drug discovery and development. The HT nature of APC that enables recordings of large datasets within a few days or even hours, makes this method ideal for applications such as: quantification of inter-patient or inter-batch variability; identification of cell sub-populations within the same batch, based on their ion channel content; and HT drug screening.

In this section we summarize the latest advances and challenges toward making such APC measurements possible.

### Advances and Recent Developments

**FIGURE 3:**
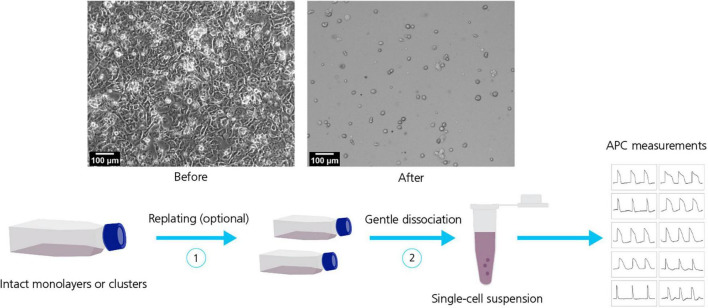
Dissociation of hiPSC-derived cell lines. Top row: Micrographs of hiPSC-CMs before (left) and after (right) dissociation. Bottom row: Dissociation of hiPSC-derived cell lines typically include a sequence of steps for example replating (1) followed by gentle dissociation methods (2) to obtain a homogeneous, healthy single-cell suspension suitable for APC measurements. Adapted from Rajamohan et al. (2016), with permission under Creative Commons Attributions License (CC BY).

Electrophysiological methods, such as MPC, have been extensively employed for characterization of hiPSC-derived cell lines over the years (Chen et al., 2014; Jones et al., 2014; Lemoine et al., 2017; Giannetti et al., 2021). However, the inherently labor intensive and low throughput nature of MPC, lead electrophysiologists to consider more time and resource efficient alternatives. Consequently, increasing efforts have gone into transferring MPC experimentation and measurements to APC systems (Haythornthwaite et al., 2012; Franz et al., 2017; Li et al., 2019; Jung et al., 2022). A key element that allows this transfer from MPC to APC is the provision of unicellular cell suspensions (see Figure 3). The quality (homogenous, unicellular populations) and health (minimal enzymatic and mechanical membrane damage and cytotoxicity) of cells in suspension are critical to achieving high success recording rates on APC. Using dissociated cell suspensions literally turns the ion channel current recording format on its head: in MPC the recording electrode is moved to an adherent cell, contrasting with APC where dissociated, single cells in suspension are moved by robotic pippetors to arrays of wells containing fixed, planar recording electrodes.

Initial with hiPSC-derived cell lines focused on APC assay development, including cell-handling on the machine; intra- and extra-cellular recording solutions; and, the suction protocols employed to position the cell and rupture the membrane, the so-called whole-cell conformation, to allow electrical access to the interior of the cell. However, progress has been slow, with only minor improvements in experiment success rate and reproducibility. It has become increasingly evident that working with hiPSC-derived cell lines demands a greater understanding of the cell population than working with clonal, homogenous immortalized cell lines. This includes the process of generating hiPSC-derived cell lines (as discussed in the section above), characterization and quality control of the final product and specialized dissociation methods for generating purer, more homogenous cell suspensions.

Whilst measurements on hiPSC-derived cell lines have progressed slowly, the technological development of APC features have advanced at a faster pace. This includes: the exchange of external and internal solutions; fast compound addition and wash out; perforated patch methods (Rosholm et al., 2021a); current clamp and dynamic clamp recordings, e.g., enabling measurements of action potentials (Becker et al., 2020; Rosholm, 2021); and temperature control for physiologically relevant temperature experimentation; all in a high throughput fashion that provides the statistical power necessary for drawing accurate and correct conclusions (Bell and Dallas, 2018; Bell and Fermini, 2021).

One major difference of APC compared to other methods typically used for hiPSC measurements, such as MPC, multielectrode-array (Odawara et al., 2016) or fluorescent plate readers (Sube and Ertel, 2017; Kilfoil et al., 2021), is that APC measures single cells that have been dissociated from 2D or 3D tissue culture. Whilst 2D/3D multi-cell or tissue recordings provide information on tissue/population behavior (including, e.g., morphology changes and network signaling), single cell APC recordings have increased temporal resolution providing crucial information regarding inter-cell variability in, e.g., compound sensitivity, different ion channel expression levels and biological activity. It is imperative to be aware of and understand which questions can be answered using dissociated cells, because several important properties such as cell morphology and ion channel expression might change due to cell handling and dissociation from culture flasks (Raman and Bean, 1997). To fill this potential information gap that can arise in cell dissociated suspensions used in APC, a complementary technology would be single cell dye-based imaging of adherent cell populations (e.g., see Vala Sciences), though this, too, has its limitations: the dye(s) used can introduce new, non-biological variables to the imaging measurements and is an indirect, proxy measure of the ion channel activity.

The preferred harvest protocol will depend on the cell type, quality and culturing days and will likely evolve continuously (also, see Figure 3). Dissociating hiPSC-derived cell lines into single-cell suspensions is often challenging (especially CMs and neurons), due to the formation of tight cellular “tissue”-like structures and networks that must be disrupted. In general, the longer the cells have been cultured, the tighter the connections become, increasing the possibility of damage during cell harvest. For APC it is of utmost importance to work with a healthy, homogenous cell suspension that is free of debris. Improvements involve: using gentle dissociating enzymes (e.g., TrypLE™, Gibco, United States; Accutase™, Stemcell Technologies, United States; or Papain, Sigma-Aldrich, United States); cold-harvest and/or incubation; addition of protective chemicals to ensure membrane integrity (e.g., ROCK inhibitor, blebbistatin) and prevent stickiness (DNase); replating for 24 h – 1 week; filtering to remove aggregates and possibly positive selection based on surface markers (Rajamohan et al., 2016; Obergrussberger et al., 2017; Li et al., 2021b). Finally, the use of fluoride containing seal enhancing solutions on many APC platforms can be problematic for some measurements, e.g., cardiac action potentials, that rely on physiologically accurate calcium kinetics. Consequently, where possible, these measurements should be performed on systems using physiological recording solutions (Mann et al., 2019; Rosholm, 2021).

Even with the aforementioned challenges, the field is now rapidly advancing and with the existing cell quality and suspension preparation methods researchers have made significant progress in exploring and developing the possibilities of measuring hiPSC-derived cell lines using APC (Rajamohan et al., 2016; Mann et al., 2019; Potet et al., 2020; Toh et al., 2020; Li et al., 2021a).

## Novel Applications of Automated Patch Clamp and Human Induced Pluripotent Stem Cell Derived Cell Lines

Originally hiPSC-derived cell lines were mainly considered for applications in regenerative medicine (Laustriat et al., 2010; Doss and Sachinidis, 2019) but their extensive range of advantages have also made them interesting for the development of cell-based assays in the fields of drug-discovery, pharmaceutical research and personalized medicine (Yu et al., 2007; Di Baldassarre et al., 2018; Berry et al., 2019). Due to the improved quality and cell handling methods, ongoing development of APC features, and initiatives such as the Comprehensive *in vitro* Proarrhythmia Assay for cardiac ion channel safety pharmacology (Colatsky et al., 2016), the field is now making good progress developing new APC applications for the characterization and pharmacology of hiPSC-derived cell lines.

In this section we provide examples of some of these novel APC applications for studies of hiPSC-derived cell lines.

### Ion Channel Analysis (Quality Control) in Human Induced Pluripotent Stem Cell Derived Cell Lines

**FIGURE 4:**
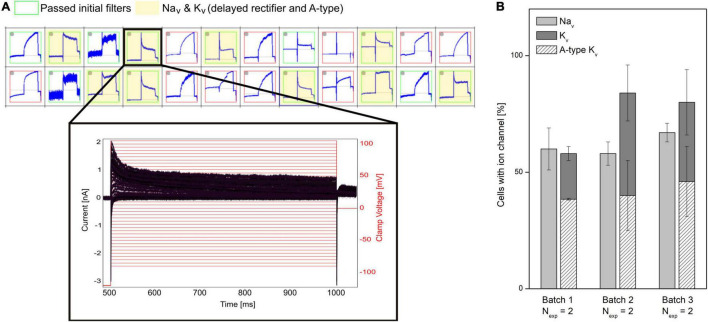
Characterizing mixed ion channel populations in hiPSC-derived cortical neurons. **(A)** Quality control filters allow selection of cells expressing the ion channels of interest. Top: Cut-out of the measurement plate view showing 24 of 384 experiments. Red and green borders correspond to experiments that failed and passed, respectively, the initial filter criteria (*R*_mem_ > 500 MΩ, *C*_cell_ > 1.5 pF). The experiments highlighted in yellow passed filter criteria for Na*_*v*_* and K*_v_* currents. Bottom: Example of a population of current traces (black) elicited by + 5 mV voltage-steps (red) recorded from a neuron, expressing Na*_*v*_* channels and a mixture of delayed rectifier and A-type K*_v_* channels. **(B)** The percentage (average ± SEM) of investigated cells containing Na*_*v*_* and K*_v_* channels were displayed for three different differentiation batches of hiPSC cortical neurons (*N*_exp_ = 2 experiment days, with > 48 measurement sites per experiment). Of the cells expressing K*_v_* channels, the percentage of K*_v_* expressing cells also having A-type K*_v_* currents are overlayed (diagonal pattern). No significant differences were observed between the three batches (Rosholm et al., 2021b). Adapted from Rosholm et al. (2021b), with permission from Sophion Bioscience A/S.

A challenge of hiPSC-derived cells is that they typically display heterogeneous current expression. This might be due to different maturation states, different cell types, genetic, and/or biological variability. In APC, currents are recorded in parallel from many cells. This makes APC well suited to characterize cell populations and identify subpopulations (Haythornthwaite et al., 2012; Franz et al., 2017). A representative example is shown in Figure 4 in which a subpopulation of hiPSC-derived cortical neurons is selected that expresses both Na*_*v*_* and a mixture of delayed rectifier and A-type K*_v_* channels. Properties of hiPSC-neurons, such as ion channel expression, AP firing characteristics and the resting membrane potential (RMP), have shown to approach that of mature adult neuronal cells by increased time in culture (Prè et al., 2014; Berry et al., 2015; Odawara et al., 2016). APC recordings can assist the evaluation of neuron population purity and maturity by comparing ion channel expression at different culturing time points or across different batches (see Figure 4).

### Action Potential Measurements in Human Induced Pluripotent Stem Cells-Derived Cardiomyocytes and Neurons

**FIGURE 5:**
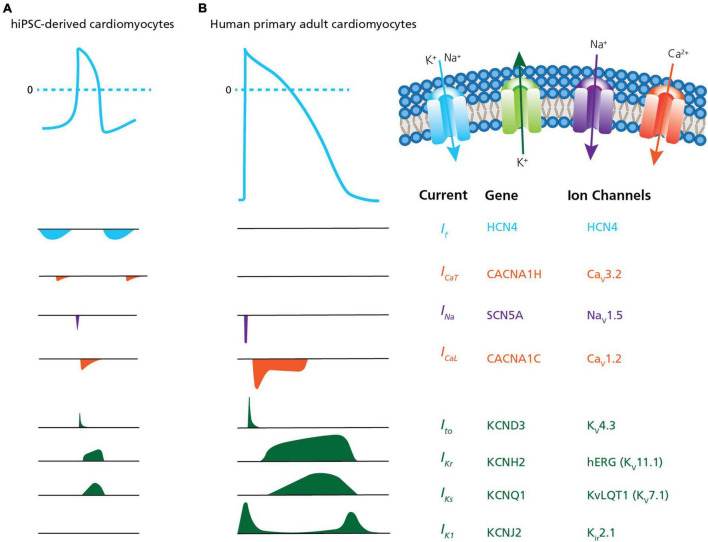
Ion channel content in hiPSC-CMs versus human primary CMs. A comparison of action potential and ion channel currents recorded in **(A)** hiPSC-CMs and **(B)** human primary adult CMs. The associated genes and ion channels are listed on the right. From the top the currents are: *I*_f_, pacemaker or funny current; *I*_CaT_, T-type calcium channel current; *I*_Na_, sodium current; *I*_CaL_, L-type calcium channel current; *I*_to_, transient outward potassium current; *I*_Kr_, rapid delayed- rectifier potassium current; *I*_Ks_, slow delayed-rectifier potassium current; and *I*_K1_, inward-rectifier potassium current. The traces highlight differences traditionally observed in hiPSC-CMs, such as the presence of the pacemaker current *I*_f_ (routinely expressed in immature CMs and lost in adult CMs) and reduced densities of hyperpolarizing current *I*_K1_. Adapted from Karbassi et al. (2020), with permission from publisher Springer Nature.

Measurements of cardiac APs are of special interest for disease modeling, drug discovery and cardiac safety (Braam et al., 2009; Mummery, 2018; Rowe and Daley, 2019; Bell and Fermini, 2021). However, the immature phenotype and heterogeneous ion channel expression of hiPSC-derived CMs, as compared to acutely isolated, primary CMs has limited the physiological relevance of their electrophysiological characterization, in general, and high throughput measurements, in particular (Casini et al., 2017; Mann et al., 2019; Vaidyanathan et al., 2021). This includes the presence of the pacemaker current *I*_*f*_ (routinely expressed in immature CMs and lost in adult CMs) and reduced densities of hyperpolarizing current *I*_*K*1_ (Jonsson et al., 2012; Goversen et al., 2018b). Highlighting these differences, Figure 5 provides a comparison between hiPSC-derived CMs and acutely isolated, primary adult CMs showing the relative size and contribution of ion channel currents that form the AP in each.

Different methods have been employed to compensate for missing currents. For example, a method that is increasingly being used is enhancing missing currents using viral transfection (Jones et al., 2014; Vaidyanathan et al., 2021). Another strategy that has been employed in APC is dynamic clamp, which is a method that models and electronically introduces ion currents into cells, to compensate for the lack of expression (Becker et al., 2013; Verkerk et al., 2017). For instance, this electronic compensation can be used to add the missing hyperpolarizing current *I*_*K*1_, bringing the depolarized RMP often observed in dissociated hiPSC-CMs closer to the physiological, hyperpolarized potential, which results in AP durations and waveforms comparable to mature CMs (Li et al., 2019; Becker et al., 2020). Drawbacks of dynamic clamp are that the compensating current is only as accurate as the assumption-based model of the mature CM, and ideally each cell’s current phenotype and derived model would need to be measured and performed individually with real-time compensation during experimentation, a potential computer processing bottleneck for the ms temporal resolution of 100 s of simultaneous recordings in APC.

**FIGURE 6:**
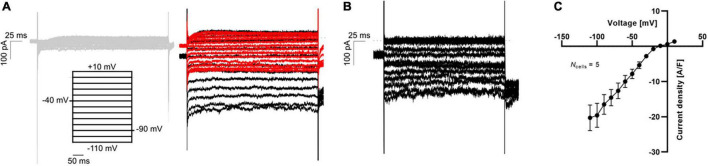
APC measurements of *I*_K1_ current-voltage relationship in hiPSC-CMs using physiological solutions. **(A)** Representative K^+^ current traces in response to a voltage step protocol from –110 mV to +10 mV with 10 mV increment step size (black). The current was measured in 5 mM extracellular K^+^ (gray, left) and 75 mM extracellular K^+^ before (black) and after (red) addition of 1 mM BaCl_2_ (right). **(B)** The BaCl_2_ sensitive *I*_K1_ was calculated by subtracting the insensitive current (red trace in **A**) from the total *I*_K_ (black trace in **A**). **(C)** The *I*_K1_ current-voltage relationship was plotted by extracting the average steady-state sensitive current (shown in **B**) and plotting as a function of step voltage. Adapted from Rosholm et al. (2022), with permission from Sophion Bioscience A/S.

Finally, the maturation of hiPSC-CMs has improved recently, and the paradigm of impaired *I*_*K*1_ expression in hiPSC-CMs has been challenged (Horváth et al., 2018). In Figure 6 we display APC measurements of “mature” *I*_*K*1_ currents in hiPSC-CMs.

As observed with hiPSC-CMs, hiPSC-neurons (including several subtypes, such as motor neurons and sensory neurons) do not completely replicate the adult neuron phenotype in terms of morphology, electrical function, and critically, in resembling neurodegenerative disease features (Casalia et al., 2021; Giacomelli et al., 2022). Although iPSC-neurons fail to entirely capture all the characteristics of fully mature brain cells, they express functional ion channels and are capable of displaying primary neuronal functions such as firing action potentials (Franz et al., 2017). Recent developments of more sophisticated and reproducible differentiation protocols combined with advanced genome engineering, longer maturation in culture and improved dissociation procedures have improved iPSC-neurons which now constitute a promising cell model for APC recordings despite their relatively immature phenotype (Shi et al., 2017; Giacomelli et al., 2022).

**FIGURE 7:**
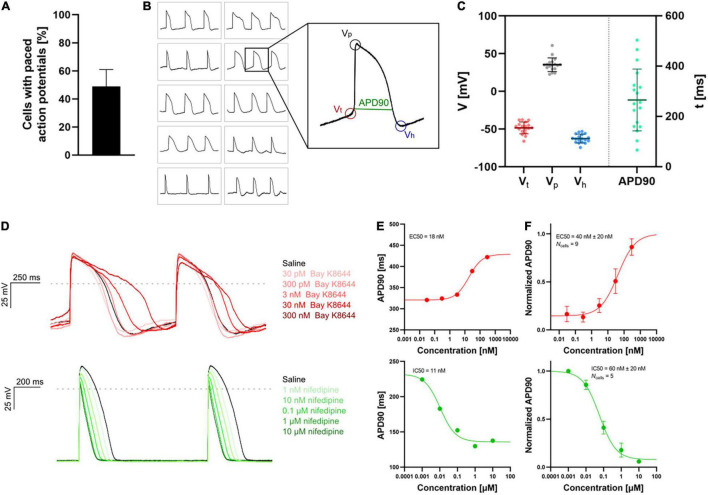
APC current-clamp measurements of paced action potentials and APD90 concentration-response plots. **(A)** The percentage of successful experiments displaying paced action potentials [*V*_*p*_ > 0 mV, resting membrane potential (RMP) < –40 mV]. Data is avg ± SD of three measurement plates (each of 48 recording sites). Assay success rate was 10%–30% out of 48 sites. **(B)** Paced action potentials from 10 individual hiPSC-CMs within a single measurement plate (left) and an expanded action potential displaying the extracted parameters: threshold potential (*V*_*t*_), peak potential (*V*_*p*_), hyperpolarization potential (*V*_*h*_), and action potential duration at 90% repolarization (APD90). **(C)** Plot of extracted parameters, *V*_*t*_ (red), *V*_*p*_ (gray), *V*_*h*_ (blue), and APD90 (green, ms) for 18 individual hiPSC-CMs with the avg ± SD (solid lines). **(D)** Paced action potentials for saline controls (black traces) and in response to increasing concentrations of Bay K8644 (top, traces in shades of red) and nifedipine (bottom, traces in shades of green). **(E)** Plots of APD90 versus compound concentration with Boltzmann fits including EC50 or IC50 calculated values, recorded in a single cell. **(F)** Cumulative concentration-response (average, APD90 normalized to pre compound concentration) relationships with Boltzmann fits including EC50 or IC50 calculated values. Data points are avg ± SEM of *N*_cells_. Adapted from Rosholm (2021) and Rosholm et al. (2022), with permission from Sophion Bioscience A/S.

The high throughput of APC allows sufficient data generation to accurately define heterogeneous ion channel populations in hiPSC-CMs, correlating channel levels to the resulting AP firing properties (Rosholm, 2021). Presently, optimized hiPSC-CM maturation and harvest protocols have made it possible to measure APs and capture compound effects in physiological solutions using APC (see Figure 7). The data were very similar to data obtained with a complementary optical assay (Seibertz et al., 2020). These advances hold great promise that robust and consistent AP assays for cardiac safety and compound screens will soon be feasible and routine.

### Disease Modeling, Drug-Screening, and Personalized Medicine

As the quality of hiPSC-derived cell lines increases, an obvious next step will be to utilize the HT capability of APC for studying disease models, drug-screening and personalized (or stratified) medicine (Chen et al., 2016). hiPSC disease models, characterization and drug-screening assays employing these cells have been developed for a range of diseases including neurodegenerative diseases (Kim, 2015; Lee and Huang, 2017) such as Alzheimer’s Disease (Medda et al., 2016; Brownjohn et al., 2017), and cardiovascular diseases (Braam et al., 2009; Sube and Ertel, 2017; Mummery, 2018; Wu et al., 2020) such as long-QT syndrome (Friedrichs et al., 2015; Jouni et al., 2015; Chen et al., 2016).

**FIGURE 8:**
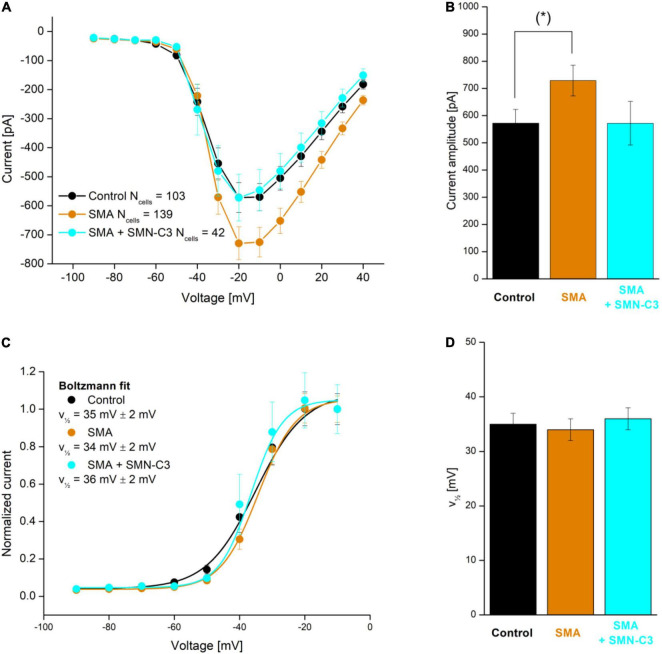
APC evaluation of compound effects on Na*_*v*_* channel properties in SMA hiPSC-neurons. Parallel recordings of control neurons (black), SMA neurons (orange), and SMA neurons treated with SMN-C3 during culturing (blue). **(A)** Average Na*_*v*_* current versus step voltage. **(B)** Quantification of the current amplitude at –20 mV. The current amplitude is significantly larger in SMA cells than in control cells [Student’s *t*-test, *p* < 0.05 (*), 95% confidence interval]. **(C)** Average Na*_*v*_* current normalized to the current recorded at V = –20 mV. Fitting of a Boltzmann function (solid lines) yielded the half-activation voltage ($V_{\frac{1}{2}}$, mV). **(D)** Half-activation voltage ($V_{\frac{1}{2}}$) values. Error bars are SEM. Adapted from Rosholm (2019), with permission from Sophion Bioscience A/S.

Although examples of APC applications within personalized biomedicine are still scarce, their need and value mean they are in active development (Friedrichs et al., 2015; Goversen et al., 2018a), with expectations high that we will see more in the near future. For an example of such an application see Rosholm (2019) in which an hiPSC motor neuron disease model, derived from a Spinal Muscular Atrophy patient (Faravelli et al., 2015) was characterized together with control cells from healthy subjects. The results showed the expected overactivity of Na*_*v*_* channels, which could be rescued by a Na*_*v*_* antagonistic compound addition (SMN-C3), see Figure 8.

## Future Perspectives

As highlighted in this review, the advances made in our labs and amongst APC and stem cell researchers across the world in the last few years have catapulted this niche, often challenging, area of research into the limelight, and is becoming widely adopted amongst the stem cell and drug discovery communities (Rajamohan et al., 2016; Franz et al., 2017). With experimental success rates climbing, improved protocols and assays developed using physiological recording conditions, the large, objective data sets generated are furthering our understanding of ion channel physiology and pathophysiology. We believe these advances in APC *in vitro* electrophysiology, married with cross-correlated combinations of animal and human iPSC models, in time will reduce and circumvent the expensive use (and sometimes limited translatability) of primary animal organ, tissue and cell models (Ebert et al., 2012; Deng, 2017; Pessôa et al., 2020). The advances made to date are providing tools and assays for significant progress in the accuracy and efficiency of safety pharmacology, drug discovery and development, and regenerative and personalized medicines.

Jumping into the fabled time traveling DeLorean car and heading back to the future: one area that is likely to feature heavily in the future of stem cell and ion channel research are the possibilities afforded by patient-derived hiPSCs. Measuring and defining an individual patient’s hiPSCs (such as their ion channel composition, pharmacology and biophysics) will allow the design of personalized, targeted medical interventions (Matsa et al., 2016; Hamazaki et al., 2017; Doss and Sachinidis, 2019), thus improving the efficacy of medicinal regimes and limiting side-effects.

Taking this concept of measuring and defining patient hiPSCs a step further: by identifying patient genotypes (by gene or genome sequencing) involved in a panel of potential health issues specific to the patient, these can be correlated to ion channel phenotypes determined using APC recordings of patient-derived hiPSCs, across a range of cell types. Such patient genotype/phenotype correlation (“phenotypic screening”) will allow existing health issues to be identified and addressed (Muñoz-Martín and Matsa, 2021). Combining this ion channel phenotypic screening with other typical phenotype screens (such as defining patient metabolic pathways or “metabolome”) will allow potential future health issues to be flagged, enabling pre-emptive, prophylactic medical interventions or lifestyle changes to be implemented. In addition, high throughput APC would allow for the application of artificial intelligence (or machine learning) tools to study multidimensional high-volume data for particular phenotypes, which may allow for identification of novel cellular biomarkers as a basis for robust drug discovery research. With time and concomitant “big data” analyses, the correlation between genotype and phenotype is likely to become sufficiently predictive allowing gene sequencing alone to be used in forecasting the ion channel targeting medicinal regimes that best suit a designated genotype patient cohort or sub-group within the general population.

In conclusion, the marriage of hiPSC and APC offers much potential for ion channel biomedical research and drug discovery. The possibilities of defining ion channel activity, biophysics and pharmacology in specialized cells (like CMs and neuronal cells), including cells derived from healthy or diseased patients, provide model cells to efficiently perform APC driven *in vitro* studies and high throughput screens that increasingly translate to human clinical studies (Rowe and Daley, 2019; Muñoz-Martín and Matsa, 2021). In writing this review, the authors aim to convey the recent adventures and advances in cellular time travel and the vast potential for these techniques and technologies. After reading, we hope the reader, like us, is now flexing their muscles in the starting blocks for the bright biomedical future of the brave new world we are soon to be sprinting toward.

## Author Contributions

KR structured the manuscript and coordinated the writing process, and produced the experimental data included in the manuscript. KR, BB, and DB prepared the first version of the manuscript. SK, DN, NV, and FS reviewed the manuscript. All authors contributed to the article and approved the submitted version.

## Conflict of Interest

KR, BB, SK, and DB are employed by Sophion Bioscience A/S, Denmark. DN was employed by Sophion Bioscience Inc., United States. The remaining authors declare that the research was conducted in the absence of any commercial or financial relationships that could be construed as a potential conflict of interest.

## Publisher’s Note

All claims expressed in this article are solely those of the authors and do not necessarily represent those of their affiliated organizations, or those of the publisher, the editors and the reviewers. Any product that may be evaluated in this article, or claim that may be made by its manufacturer, is not guaranteed or endorsed by the publisher.

## Abbreviations

APs: action potentials
APC: automated patch clamp
CMs: cardiomyocytes
hiPSC: human induced pluripotent stem cell
HT: high throughput
MPC: manual patch clamp
PSCs: pluripotent stem cells.
